# Comparative Effectiveness of Epidural Analgesia and Intravenous Lidocaine for Postoperative Pain in Major Abdominal Surgery: A Systematic Review and Meta-Analysis

**DOI:** 10.1155/anrp/9822744

**Published:** 2025-02-28

**Authors:** Mohammad Jawwad, Dawar Nadeem Aslam Dar, Rana Faheem Ullah Khan, Aizaz Chaudhry, Faraz Arkam, Asad Gul Rao, Yusra Mir, Mohammad Maheer Mubashir, Aqsa Mir, Haider Imran, Umar Maqbool, Pierina Clementine Pereira

**Affiliations:** ^1^Department of Internal Medicine, Dow University of Health Sciences, Karachi 74200, Pakistan; ^2^Department of General Surgery, Services Institute of Medical Sciences, Lahore 54610, Pakistan; ^3^Department of Internal Medicine, Shaikh Khalifa Bin Zayed Al-Nahyan Medical and Dental College, Lahore 54600, Pakistan; ^4^Department of General Surgery, Quaid-e-Azam Medical College, Bahawalpur 63100, Pakistan; ^5^Department of Neurosurgery, Shifa Tameer-e-Millat University, Islamabad 46000, Pakistan; ^6^Department of General Surgery, SMBBMU Chandka Medical College, Larkana 77170, Pakistan; ^7^Deparment of General Surgery, King Edward Medical University, Lahore 54000, Pakistan; ^8^Department of General Surgery, Liaquat National Hospital and Medical College, Karachi 74800, Pakistan; ^9^Department of Internal Medicine, Foundation University Medical College, Islamabad 44000, Pakistan; ^10^Department of Internal Medicine, Jinnah Sindh Medical University, Karachi 75550, Pakistan

**Keywords:** analgesia, lidocaine, morphine, pain management, postoperative pain

## Abstract

**Background:** Pain management is an integral part of recovery after major abdominal surgeries. Traditionally, epidural analgesia is used for postoperative pain management in major abdominal surgeries. However, intravenous lidocaine has recently been proven to be a good alternative. However, there is very limited evidence comparing their efficacy in major abdominal surgery. The aim of this review is to compare the effectiveness of epidural analgesia with intravenous lidocaine in reducing pain and opioid consumption following major abdominal surgery.

**Methods:** We searched PubMed and Cochrane Library from inception to May 2024 to identify studies that match our topic. We performed all statistical analyses using RevMan. The primary outcome was pain scores. The other outcomes were opioid requirements, postoperative nausea and vomiting, hospital stay duration, and time to pass flatus.

**Results:** Seven studies (six randomized clinical trials and one observational study; *n* = 643) were included. Our results suggest that epidural bupivacaine significantly reduced pain scores during the first 24 h postoperatively as compared with the patients who received intravenous lidocaine (Std. mean difference: −0.23; 95% confidence interval [CI]: −0.40, −0.06; and *p*=0.008). There was no difference at 48 h (Std. mean difference: −0.09; 95% CI: −0.27, 0.08; and *p*=0.028) and 72 h intervals (Std. mean difference: −0.08; 95% CI: −0.25, 0.09; and *p*=0.037).

**Conclusion:** Our study shows that epidural analgesia, particularly epidural bupivacaine, provides superior pain relief as compared to intravenous lidocaine during the first 24 h postoperatively. However, there was heterogeneity among studies. Thus, in future, large standardized randomized controlled trials are required.

## 1. Introduction

Postoperative pain is a common problem within the first 24 h after surgery, with 22%–67% of the patients experiencing pain [1–3]. Hence, effective pain management is crucial as it impacts patient outcomes such as morbidity, hospital stay duration, and patient satisfaction [4]. This is also true for major abdominal surgeries, which are quite complex procedures that require effective postoperative analgesia to ensure better recovery of the patients.

Epidural analgesia and intravenous lidocaine are some of the methods used for managing postoperative pain in major abdominal surgeries. Intravenous lidocaine which is a local anesthetic works by blocking the conduction of pain signals in the spinal cord [5]. In contrast, epidural analgesia provides targeted pain relief at the surgical site through the administration of local anesthetics or opioids via a catheter placed in the epidural space [6].

Each method has its own advantages and disadvantages, and the choice between the two is often guided by the patient-specific factors, surgeon preferences, and institutional protocols.

Epidural analgesia, which is quite effective in managing postoperative pain, is used in approximately 50%–60% of abdominal surgeries worldwide for postoperative pain management [7]. However, it is also invasive, time consuming, expensive, and requires a high level of clinical skill [8, 9]. In addition, epidural analgesia has reported high failure rates of 10%–30% [10] and contraindications such as hemodynamic instability, increased intracranial pressure, and local infection at the puncture site [6]. Owing to these limitations, alternative treatment options such as pregabalin, clonidine, and intravenous lidocaine have been explored for their potential to be utilized as a postoperative analgesic.

However, only a few studies have been found in the literature who have compared the efficacy of intravenous lidocaine with epidural analgesia in major abdominal surgeries and the results of these studies are conflicting, making it difficult to decide between the two interventions for postoperative pain management in major abdominal surgeries. Therefore, a meta-analysis has been conducted to compare the efficacy of these two interventions. The primary outcome used for comparison in our study is mean pain scores at different time intervals. The secondary outcome is daily morphine consumption. Additional outcomes are postoperative nausea and vomiting, mean hospital stay duration, and mean time to pass flatus.

## 2. Methods

### 2.1. Data Sources and Search Strategy

We employed the Preferred Reporting Items for Systematic Reviews and Meta-Analyses 2020 (PRISMA 2020) statement for this meta-analysis, the checklist of which is given in Supporting Table 1 [11]. The PubMed and Cochrane Library were used to search and conduct comprehensive reviews of the relevant articles from their inception up to May 2024. The detailed search strategy is given in Supporting Table 2. The reference lists of the retrieved articles and previous meta-analyses that might be relevant to our topic were then reviewed manually. Also our study is registered with the International Prospective Register of Systematic Reviews (PROSPERO) with registration number CRD42024561495.

### 2.2. Study Selection and Eligibility Criteria

Two reviewers (D.N.A.D. and R.F.U.K.) independently reviewed and selected the articles based on the eligibility criteria. A third reviewer (F.A.) was consulted in the event of disagreements. All the articles obtained through search strategy were exported to EndNote Reference Library (Version 21), where duplicates were determined and eliminated. We included six randomized controlled trials and one observational study in our meta-analysis that compared the efficacy of epidural analgesia with intravenous lidocaine for postoperative pain management in the major abdominal surgery. Patients greater than 18 years of age were included in our study. The following studies were excluded: (a) studies not including the mean pain scores at rest for comparison; (b) studies including patients having surgeries other than major abdominal surgeries; (c) studies not comparing epidural analgesia with intravenous lidocaine; (d) studies having insufficient data; (d) duplicate studies or overlapping participants; (e) systematic reviews, meta-analysis, pilot studies, editorials, conference papers, case reports, or animal experiments; and (f) studies not published in the English language. The primary outcome of our study is mean pain scores at rest. The secondary outcome is daily morphine consumption. Additional outcomes are postoperative nausea and vomiting, mean hospital stay duration, and mean time to pass flatus.

### 2.3. Data Extraction and Quality Assessment

The following data were obtained from the included studies: (1) study characteristics such as first author, publication year, and study design; (2) methods such as dosage and type of anesthetic agent used; (3) study population characteristics such as sample sizes, mean age groups, and gender; (4) primary outcome, that is, mean pain scores at rest at different time intervals; (5) secondary outcome, that is, daily morphine consumption; (6) incidence of postoperative nausea and vomiting; (7) mean hospital stay duration in days; and (8) mean time to pass flatus in hours.

The primary outcome was analyzed using mean and standard deviation as parameters of interest. The study data under analysis were presented in either tabulated or graphical form. The data was given in a tabulated form by Casas-Arroyave, Osorno-Upegui, and Zamudio-Burbano [12], Staikou et al. [13], and Wongyingsinn et al. [14]. The pain scores at rest were reported with mean values and standard deviations on numeric pain rating (NPR/numeric rating scale [NRS]) scale by Casas-Arroyave, Osorno-Upegui, and Zamudio-Burbano [12], and Staikou et al. [13] whereas in Wongyingsinn et al.'s study [14], the data were reported on the verbal rating scale (VRS) with median and interquartile ranges as parameters based on the site of abdominal surgery in two subgroups of patients. Studies that reported their findings as median and interquartile ranges were converted into mean and standard deviation using normal-based methods for data transformation devised by Luo et al. [15] and Wan et al. [16]. In the case of Wongyingsinn et al. [14], the data from both subgroups were converted into their means and standard deviations separately before combining the parameters from both subgroups to attain pooled mean and standard deviation for each arm [17].

Kuo et al. [18], Swenson et al. [19], Terkawi et al. [20], and Jayaprabhu et al. [21] presented their data in the graphical form. Data were presented either as a bar graph or a whisker plot where the mean or median was represented as the upper limit of the vertical bar and the standard deviation or interquartile range was represented as the range of error bar or whisker abutting the upper limit, respectively. Automated visual data extraction was used to extract the data from the visually represented data while keeping the measurement scale discrepancies to a bare minimum. The data was reported as mean and standard deviation on the visual analog scale (VAS) and NRS by Kuo et al. [18] and Jayaprabhu et al. [21], respectively. Terkawi et al. [20] reported their data as mean and standard error on the NRS pain scale and by Swenson et al. [19] as the median and interquartile ranges for their pain scores. In the case of Terkawi et al. [20], the standard error was converted into the standard deviation by multiplying it with the square root of the sample size.

For daily morphine consumption, data were extracted from four studies that reported daily morphine consumption at similar intervals while other studies showed morphine consumption at different intervals, so data were not extracted from them. Also, the dosage of opioids was standardized by converting it into IV morphine in milligrams. Data extraction was carried out independently by two investigators (D.N.A.D. and R.F.U.K.) independently using the criteria mentioned above and the disagreements were resolved either by consensus or with the help of a third investigator (A.C.).

Revised Cochrane risk-of-bias tool for randomized trials and the Newcastle–Ottawa quality assessment scale were used to assess the risk of bias for the included studies. Two investigators (A.C. and A.M.) independently assessed the quality of the data, and any inconsistencies were resolved by consensus or with the help of a third investigator (D.N.A.D.) (Supporting Table 3).

### 2.4. Statistical Analysis

We performed all statistical analyses using RevMan (Version 5.4). As mean pain scores at rest were reported on different scales such as NRS, VAS, and VRS, we used standardized mean difference (Std. mean difference) with 95% confidence intervals (CIs) to calculate the continuous data as meaningful effect measures for the primary outcome. Other outcomes which include mean daily morphine consumption, mean time to pass flatus, and mean hospital stay duration had used same units; therefore, mean difference with 95% (CIs was used to calculate the continuous data as meaningful effect measures. However, for postoperative nausea and vomiting, we used risk ratio (RR) with 95% CIs to calculate the dichotomous data as meaningful effect measures. The random-effects model and Higgins *I*^2^ statistic were used to analyze and evaluate heterogeneity, respectively. We considered an *I*^2^ of < 50%, 50%–75%, and > 75% indicating low, moderate, and high heterogeneity, respectively [22]. The hypothesis was considered statistical significant if its *p* value was less than or equal to 0.05. In case of heterogeneity > 50%, we performed a sensitivity analysis to identify the study causing heterogeneity. In addition, subgroup analysis was performed based on study design (RCTs and observational studies) and the type of intervention used (epidural bupivacaine and epidural lidocaine). We did not perform any funnel plot asymmetry tests because when less than 10 papers are included in the analysis (which is the case for all outcomes in our study), Cochrane guidelines do not recommend them. In such instances, the test's power is insufficient to distinguish between random and actual asymmetry [23].

## 3. Results

### 3.1. Basic Characteristics

**Figure 1:**
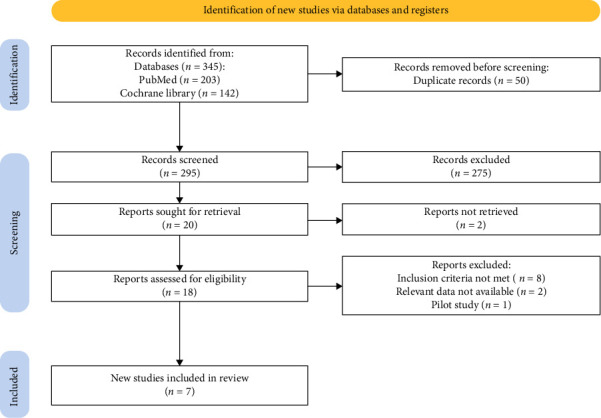
PRISMA flow diagram.

The initial search related to the topic led to 345 potential articles up to May 2024. First, 50 duplicated articles were excluded and the remaining 295 articles were screened by reading the titles and abstracts after which additional 275 articles were excluded and 20 articles were sought for retrieval. Out of these 20 full-text articles, 18 were found which were carefully screened. From these articles, 11 were excluded. Eight did not meet the inclusion criteria, two were reviews, and one was a pilot study. Finally, seven articles are included in our meta-analysis. The details are highlighted in the PRISMA flowchart (Figure 1) based on the PRISMA 2020 statement [11].

Out of seven studies, six are randomized controlled trials and one is a retrospective cohort study. These studies gave us a pool of 643 patients, out of which 318 patients were given epidural analgesia and 325 patients were given intravenous lidocaine infusion. Out of 643 patients, 328 were males and 315 were females. The overall mean age in the epidural analgesia group calculated from all studies was 62.0 while in the intravenous lidocaine group, it was 58.6. The details of the data provided in these studies and the patients' essential demographic characteristics are provided in Table 1.

For randomized controlled trials, the quality assessment was conducted according to the revised Cochrane risk-of-bias tool for randomized trials and for observational study, the Newcastle–Ottawa quality assessment scale was employed (Supporting Table 3).

### 3.2. Primary Outcome

**Figure 2:**
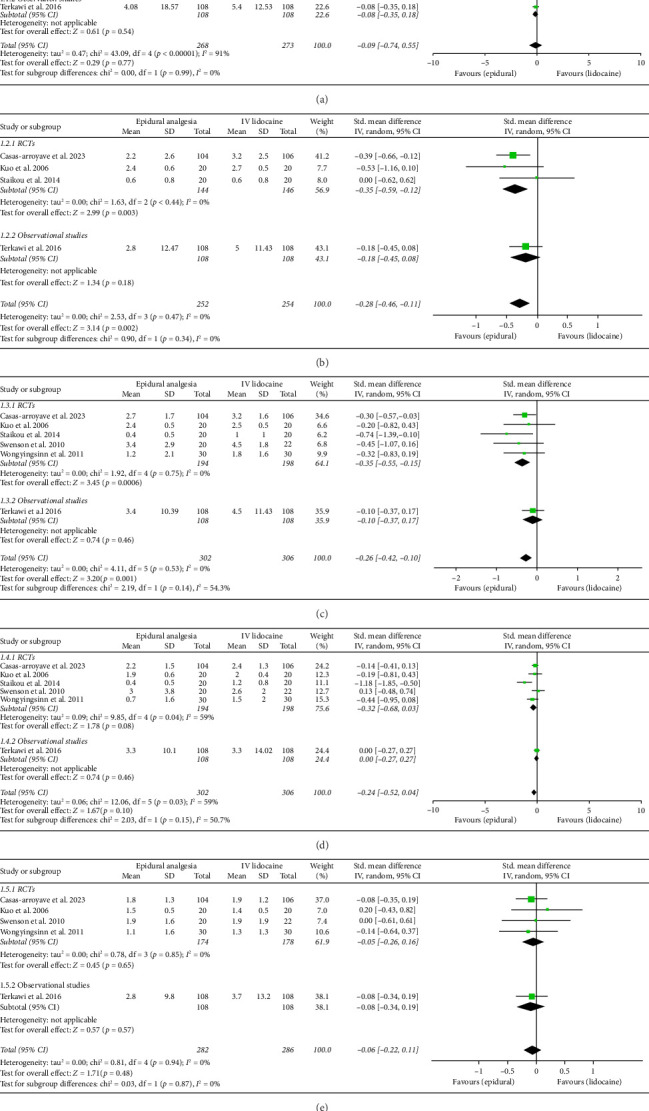
Forest plots of mean pain scores. (a) At 2 h interval. (b) At 12 h interval. (c) At 24 h interval. (d) At 48 h interval. (e) At 72 h interval.

Mean pain scores at 2-h interval (Std. mean difference: −0.09; 95% CI: −0.74, 0.55; and *p*=0.77; Figure 2(a)) were reported by five studies which showed no significant difference between the two intervention groups but the heterogeneity was high (*I*^2^ = 91%). Therefore, sensitivity analysis was employed which significantly reduced heterogeneity to 38% by removing Kuo et al. [18] and Staikou et al. [13] (Std. mean difference: −0.27; 95% CI: −0.53, 0.02; and *p*=0.04; Supporting Figure 1A) with the effect favoring epidural analgesia group significantly. RCTs and observational studies subgroups did not show any statistical difference (*p*=0.99) for mean pain scores at 2 h interval.

Mean pain scores at 12 h (Std. mean difference: −0.28; 95% CI: −0.46, −0.11; and *p*=0.002; Figure 2(b)) and at 24 h intervals (Std. mean difference: −0.26; 95% CI: −0.42, −0.10; and *p*=0.001; Figure 2(c)) were reported by four and six studies, respectively. Integrated analysis showed statistical difference between the two intervention groups with mean pain scores at both intervals favoring the epidural group and also there was low heterogeneity at both intervals (*I*^2^ = 0%). RCTs and observational studies subgroups did not show any significant difference at 12 h (*p*=0.34) and 24 h intervals (*p*=0.14).

Mean pain scores at 48 h interval (Std. mean difference: −0.24; 95% CI: −0.52, 0.04; and *p*=0.10; Figure 2(d)) were reported by six studies that showed no significant difference between the two intervention groups. The heterogeneity was moderate ((*I*^2^ = 59%) which was significantly reduced ((*I*^2^ = 0%) by performing sensitivity analysis by eliminating Staikou et al. [13] (Std. mean difference: −0.09; 95% CI: −0.26, 0.07; and *p*=0.27; Supporting Figure 1B) while the effect remained the same. Randomized controlled trials and observational studies subgroups did not show any significant difference at 48 h interval (*p*=0.15).

Mean pain scores at 72 h interval (Std. mean difference: −0.06; 95% CI: −0.22, 0.11; and *p*=0.48; Figure 2(e)) were reported by five studies that showed no significant difference between the two intervention groups. The heterogeneity was low ((*I*^2^ = 0%). A subgroup analysis between RCTs and observational studies did not show any significant difference in this case (*p*=0.87).

Subgroup analysis depending on the nature of analgesic agent used in the epidural group was done, which showed a significant decrease in mean pain scores of the epidural intervention group as compared with the IV lidocaine group in studies using bupivacaine at 2 h (Std. mean difference: −0.27; 95% CI: −0.53, −0.02; and *p*=0.04; Supporting Figure 2A), 12 h (Std. mean difference: −0.29; 95% CI: −0.49, −0.08; and *p*=0.006; Supporting Figure 2B), and 24 h intervals (Std. Mean Difference: −0.23; 95% CI: −0.40, −0.06; and *p*=0.008; Supporting Figure 2C), while there was no difference in mean pain scores in studies using Lidocaine at 2 h (Std. mean difference: 0.35; 95% CI: −2.80, 3.50; and *p*=0.83; Supporting Figure 2A), 12 h (Std. mean difference: −0.26; 95% CI: −0.78, 0.26; and *p*=0.32; Supporting Figure 2B), and 24 h intervals (Std. mean difference: −0.46; 95% CI: −1.00, 0.07; and *p*=0.09; Supporting Figure 2C). However, both epidural lidocaine and bupivacaine subgroups did not show any significant difference when compared with intravenous lidocaine at 48 h and 72 h intervals (Supporting Figures 2D and 2E).

### 3.3. Secondary Outcome

**Figure 3:**
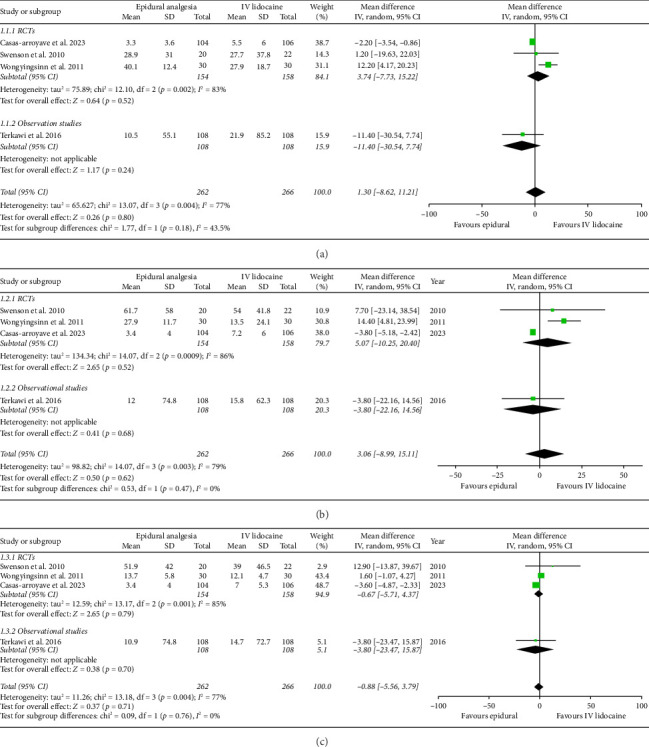
Forest plots of mean daily morphine consumption equivalent to IV mg of morphine. (a) At 24 h interval. (b) At 48 h interval. (c) At 72 h interval.

Mean daily morphine consumption at 24 h (mean difference: 1.30; 95% CI: −8.62, 11.21; and *p*=0.80; Figure 3(a)), 48 h (mean difference: 3.06; 95% CI: −8.99, 15.11; and *p*=0.62; Figure 3(b)) and 72 h intervals (mean difference: −0.88; 95% CI: −5.56, 3.79; and *p*=0.71; Figure 3(c)) were reported by four studies which showed no significant difference between the two intervention groups but the heterogeneity was high at every interval under analysis (*I*^2^ = 77%, 79%, and 77%, respectively), which was significantly reduced (*I*^2^ = 0%) by removing Wongyingsinn et al. [14] through sensitivity analysis.

However, after performing the sensitivity analysis, mean daily morphine consumption was significantly reduced in the epidural group as compared with the intravenous lidocaine group at 24 h (mean difference: −2.23; 95% CI: −3.56, −0.90; and *p*=0.001; Supporting Figure 3A), 48 h (mean difference: −3.78; 95% CI: −5.15, −2.41; and *p* < 0.00001; Supporting Figure 3B) and 72 h intervals (mean difference: −3.56; 95% CI: −4.83, −2.30; and *p* < 0.00001; Supporting Figure 3C). RCTs and observational studies subgroups did not show any statistical difference for mean daily morphine consumption at 24 h (*p*=0.47), 48 h (*p*=0.47), and 72 h intervals (*p*=0.76).

### 3.4. Additional Outcomes

**Figure 4:**
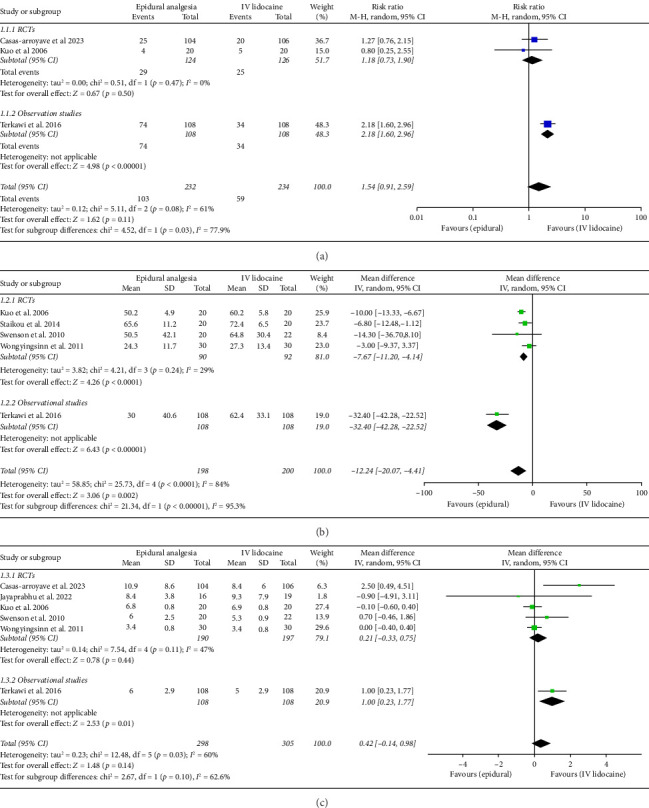
Forest plot of (a) 3 day incidence of postoperative nausea and vomiting, (b) mean time to pass first flatus in hours, and (c) mean hospital duration in days.

For the incidence of postoperative nausea and vomiting over 72 h among the patients from the two intervention groups, three studies had adequate data. Pooled analysis from these studies showed no significant difference between the two intervention groups (RR: 1.54; 95% CI: 0.91, 2.59; and *p*=0.11; Figure 4(a)). The heterogeneity was moderate (*I*^2^ = 61%). The sensitivity analysis to reduce heterogeneity could not be performed because minimum three studies are required for analysis. For RCTs and observational studies subgroups, there was significant difference (*p*=0.03).

For the comparison of mean time to pass flatus in hours between the two interventions groups, five studies had relevant data which were then analyzed. Pooled analysis from these studies showed significant difference between the two intervention groups (mean difference: −12.24; 95% CI: −20.07, −4.41; and *p*=0.002; Figure 4(b)) with the effect favoring the epidural analgesia group. The heterogeneity was high (*I*^2^ = 84%), which was significantly reduced by performing sensitivity analysis by eliminating Terkawi et al. [20] (*I*^2^ = 29%), and the effect remained the same (mean difference: −7.67; 95% CI: −11.20, −4.14; and *p* < 0.0001; Supporting Figure 4). For RCTs and observational studies subgroups, there was a significant difference (*p* < 0.00001). But overall effect (*p*=0.002) and effect in case of RCTs subgroup (*p* < 0.0001) remained the same.

Mean hospital duration in days was reported in six studies which was then analyzed. Pooled analysis from these studies showed no statistical difference between the two intervention groups (mean difference: 0.42; 95% CI: −0.14, 0.98; and *p*=0.14; Figure 4(c)). The heterogeneity was moderate (*I*^2^ = 60%) which was reduced by performing sensitivity analysis by eliminating Terkawi et al. [20] (*I*^2^ = 47%) and the effect remained the same (mean difference: 0.21; 95% CI: −0.33, 0.75; and *p*=0.11; Supporting Figure 5). RCTs and observational studies subgroups did not have significant difference (*p*=0.1).

## 4. Discussion

Our meta-analysis compared the efficacy of intravenous lidocaine with epidural analgesia for postoperative pain management in major abdominal surgery. Our study showed that patients who received epidural analgesia as compared with intravenous lidocaine have reduced mean pain scores at 12 h and 24 h intervals while there was no significant difference between the two groups at 2 h, 48 h, and 72 h intervals.

However, the heterogeneity was high at 2 h and 48 h intervals, which was significantly decreased by performing sensitivity analysis. But mean pain scores were significantly reduced in the epidural group as compared with the intravenous lidocaine group at 2 h interval while the effect remained the same at 48 h interval after sensitivity analysis. This heterogeneity could be explained by removing both Staikou et al. [13] and Kuo et al. [18] at 2 h interval and only Staikou et al. [13] at 48 h interval because in these studies, epidural lidocaine was used as anesthetic agent whereas epidural bupivacaine was used in the rest of the studies.

In addition, a subgroup analysis was performed based on the type of epidural analgesic used which showed that the patients who received epidural bupivacaine had a significant reduction in their mean pain scores in contrast to the patients who received intravenous lidocaine at 2 h, 12 h, and 24 h intervals whereas no significant reduction was observed at 48 h and 72 h intervals. On the other hand, there was no significant difference in mean pain scores between the patients receiving epidural lidocaine and those receiving intravenous lidocaine at each interval under analysis.

Hence, this study proves that the patients who received epidural bupivacaine for postoperative pain management have decreased pain scores during the first 24 h after surgery as compared with the patients who received intravenous lidocaine. However, there was no significant difference in pain scores over the next 2 days in both groups.

Our meta-analysis showed that there was no significant difference in the reduction of morphine consumption postoperatively among patients receiving epidural analgesia or intravenous lidocaine at 24 h, 48 h, and 72 h intervals.

A sensitivity analysis was employed before analyzing the outcomes to reduce the heterogeneity between the studies at 24 h, 48 h, and 72 h intervals. This heterogeneity can be attributed to Wongyngsinn et al.'s [14] study where the results were reported in the intravenous lidocaine intervention group as intravenous morphine whereas in the epidural group, it was reported as epidural morphine. However, the rest of the studies reported results as IV morphine in both intervention groups. Though conversion methods were employed, this difference could still be the cause of heterogeneity between the studies.

Thus, after decreasing heterogeneity, our meta-analysis reveals that patients receiving epidural analgesia require less morphine postsurgery for pain management as compared with the patients receiving intravenous lidocaine.

In terms of incidence of postoperative nausea and vomiting, our meta-analysis showed no significant difference between the two groups but the heterogeneity was high and only three studies were included. As minimum of three studies are required for meta-analysis, sensitivity analysis could not be performed.

Furthermore, our study showed that patients receiving epidural analgesia as compared with those receiving intravenous lidocaine took lesser time to pass flatus while there was no significant difference between the two groups in terms of mean hospital stay duration. However, the heterogeneity was high in both cases, which was significantly reduced by performing sensitivity analysis, leading to similar overall results. This heterogeneity could be attributed to Terkawi et al. [20], which is a retrospective observational study that depended upon the old patient records which could be subjected to errors.

During the literature review, two systematic reviews comparing epidural analgesia with intravenous lidocaine were found and studied in detail. However, these studies provided little insight into which modality provides the most effective pain control. One study showed that intravenous lidocaine has limited benefit in reducing early pain and morphine usage when compared with a placebo; however, there was not any significant reduction in pain or opioid consumption when compared with the epidural group [24] whereas the other study provided little evidence to prove that whether lidocaine is better than epidural analgesia in reducing pain scores [25].

From the abovementioned studies, it is found that there is a conflict in the available literature regarding the comparison of efficacy between the two intervention groups in postoperative pain management. Also, the literature comparing both groups in major abdominal surgeries is very scarce. Thus, our study tries to resolve this conflict by providing statistically significant evidence that epidural analgesia is superior to intravenous lidocaine in terms of early postoperative pain control in major abdominal surgery.

Our analysis had several limitations. First, there were only a small number of relevant trials and they were also heterogeneous. In addition, sample sizes in most of the included studies were small, which meant that there was a risk of overestimating or missing a treatment effect. There was variability in the duration of postoperative infusions. For instance, in some studies, the infusions were stopped just after the operation while in other studies, they were stopped after 24 h of surgery, which might have resulted in heterogeneity. Also, there were differences in the dosage of intravenous lidocaine in different studies ranging from 0.5 to 1 mg/min in Terkawi et al. [20] to 3 mg/kg/hour in Kuo et al. [18]. Similarly, 0.1% bupivacaine along with opioids used for epidural analgesia exhibits considerable variability across different studies in terms of their infusion rates ranging from 4 mL/hour in Jayaprabhu et al. [21] to 10 mL/hour in Swenson et al. [19]. Also, the type and dosages of adjuncts opioids varied across studies such as in Terkawi et al.'s study [20], 10 μg/mL hydromorphone was used while in Casas-Arroyave, Osorno-Upegui, and Zamudio-Burbano's study [12], 20 μg/mL of morphine was used. These differences in dosages and adjunct types could substantially impact clinical outcomes such as analgesic efficacy and the risk of side effects, including hypotension, motor blockade, and respiratory depression, leading to considerable heterogeneity among studies. The included studies did not report all of the outcomes we assessed. Also, some of the studies were high risk on quality assessment. Analysis of some of the outcomes in our study revealed high heterogeneity which was reduced significantly after sensitivity analysis was performed to identify and remove the possible causes of heterogeneity.

## 5. Conclusion

Our meta-analysis shows that epidural analgesia particularly epidural bupivacaine provides greater pain relief as compared with intravenous lidocaine at 2 h, 12 h, and 24 h intervals postoperatively. Furthermore, patients in the epidural analgesia group relied less on rescue morphine and took less time to pass flatus in comparison with the patients in the intravenous lidocaine group. However, there was no difference in hospital stay duration and incidence of postoperative nausea and vomiting between the two groups. In conclusion, epidural analgesia should be considered for optimal early postoperative pain control in major abdominal surgery after weighing its benefits of superior pain control against its procedural technicalities and potential risks associated with its administration. However, in cases where it is absolutely contraindicated intravenous lidocaine remains a good alternative. There were several limitations in our study which led to an increase in heterogeneity between the studies that may affect the robustness of our findings. Therefore, further large-scale randomized controlled trials with standardized treatment protocols are required to minimize heterogeneity in the future and to establish optimal patient-specific regimens in future.

## Figures and Tables

**Table 1:** Basic demographics of studies.

No	Study name	Published time	Study design	Intervention method used		Sample size (*n*)			Age (Mean [SD])		Sex (*n*)					
				Epidural analgesia	Intravenous lidocaine	Epidural analgesia	Intravenous lidocaine	Total	Epidural analgesia	Intravenous lidocaine	Male			Female		
											Epidural analgesia	Intravenous lidocaine	Total	Epidural analgesia	Intravenous lidocaine	Total
1	Casas-Arroyave, Osorno-Upegui, and Zamudio-Burbano [12]	2023	RCT	0.1% Bupivacaine plus 20 μg/mL of morphine at the rate of 7 mL/hr	2% Lidocaine at the rate of 1 mg/kg/hr	104	106	210	69.9 (15)	60 (16.2)	50	53	103	54	53	107
2	Jayaprabhu et al. [21]	2022	RCT	0.1% Bupivacaine plus 20 μg of fentanyl at the rate of 4–5 mL/hr	2% Lidocaine at the rate of 2 mg/kg/hr	16	19	35	51.1 (12.4)	47.7 (15.8)	11	13	24	5	6	11
3	Terkawi et al. [20]	2016	Retrospective cohort study	0.1% Bupivacaine plus 10 μg/mL of hydromorphone at the rate of 8 mL/hr	2% Lidocaine at the rate of 0.5–1 mg/minute	108	108	216	58.2 (13.7)	57.3 (14.8)	49	39	88	59	69	128
4	Staikou et al. [13]	2014	RCT	2% Lidocaine at the rate of 2 mg/kg/hr	2% Lidocaine at the rate of 2 mg/kg/hr	20	20	40	68.6 (10.8)	73.6 (7.5)	16	12	28	4	8	12
5	Wongyingsinn et al. [14]	2011	RCT	0.1% Bupivacaine plus 0.02 mg/mL of morphine	2% Lidocaine at the rate of 1 mg/kg/hr	30	30	60	61 (15)	58 (16)	19	19	38	11	11	22
6	Swenson et al. [19]	2010	RCT	0.1% Bupivacaine plus 6 μg/mL of hydromorphone at the rate of 10 mL/hr	2% Lidocaine at the rate of 2 mg/minute	20	22	42	46.1 (14.4)	51.3 (17.4)	16	10	26	4	12	16
7	Kuo et al. [18]	2006	RCT	2% Lidocaine at the rate of 3 mg/kg/hr	2% Lidocaine at the rate of 3 mg/kg/hr	20	20	40	61.6 (22.3)	62.6 (19.5)	11	10	21	9	10	19

*Note:* Here, data are presented in ‘*n*' which is frequency for sample size and sex and presented in mean (standard deviation) for age.

## Data Availability

The data that support the findings of this study are available from the corresponding author upon reasonable request.

## Supporting Information

Supporting Information Supporting Table 1: PRISMA 2020 Checklist.

Supporting Information Supporting Table 2: Search strategy.

Supporting Information Supporting Table 3: (A) RCTS: Quality Assessment according to Revised Cochrane risk-of-bias tool for Randomized Trials (ROB2). (B) Observational Study: Quality Assessment according to Newcastle–Ottawa scale.

Supporting Information Supporting Figure 1: Forest Plots of Mean Pain Scores (Sensitivity Analysis). (A): At 2 h Interval. (B): At 48 h Interval.

Supporting Information Supporting Figure 2: Forest Plots of Mean Pain Scores (Subgroup Analysis: Epidural Bupivacaine vs. Epidural Lidocaine). (A): At 2 h Interval. (B): At 12 h Interval. (C): At 24 h Interval. (D): At 48 h Interval. (E): At 72 h Interval.

Supporting Information Supporting Figure 3: Forest Plots of Mean Daily Morphine Consumption equivalent to IV mg of morphine (Sensitivity Analysis). (A): At 24 h Interval. (B): At 48 h Interval. (C): At 72 h Interval.

Supporting Information Supporting Figure 4: Forest Plots of Mean time to pass first flatus in hours (Sensitivity Analysis).

Supporting Information Supporting Figure 5: Forest Plots of Mean Hospital Duration in days (Sensitivity Analysis).
