# Supplementary material for: Comparative Effectiveness of Epidural Analgesia and Intravenous Lidocaine for Postoperative Pain in Major Abdominal Surgery: A Systematic Review and Meta-Analysis
Source: Anesthesiol Res Pract. 2025 Feb 28;2025:9822744. doi: 10.1155/anrp/9822744 (PMC11991782; doi:10.1155/anrp/9822744)
Supplement: Supporting Information — Supporting Table 2: Search strategy. [file 9822744.f2.docx]

| Database | Strings | Results |
| --- | --- | --- |
| PubMed | IV Lidocaine OR Intravenous Lidocaine AND Epidural Analgesia | 203 |
| Cochrane Library | IV Lidocaine versus Epidural Analgesia OR Intravenous Lidocaine vs Epidural Analgesia OR IV Lidocaine vs Epidural Analgesia OR Intravenous Lidocaine versus Epidural Analgesia | 142 |

**Supplemental Table 2:** Search strategy
