# Supplementary material for: Comparative Effectiveness of Epidural Analgesia and Intravenous Lidocaine for Postoperative Pain in Major Abdominal Surgery: A Systematic Review and Meta-Analysis
Source: Anesthesiol Res Pract. 2025 Feb 28;2025:9822744. doi: 10.1155/anrp/9822744 (PMC11991782; doi:10.1155/anrp/9822744)
Supplement: Supporting Information — Supporting Table 3: (A) RCTS: Quality Assessment according to Revised Cochrane risk-of-bias tool for Randomized Trials (ROB2). (B) Observational Study: Quality Assessment according to Newcastle–Ottawa scale. [file 9822744.f3.docx]

| **Study Name** | **Domain 1** | **Domain 2** | **Domain 3** | **Domain 4** | **Domain 5** | **Overall** | |
| --- | --- | --- | --- | --- | --- | --- | --- |
| Casas-Arroyave et al 2023 | Low Risk | Low Risk | Low Risk | Low Risk | Some Concerns | Some Concerns | |
| Jayaprabhu et al 2022 | Low Risk | Low Risk | Low Risk | High Risk | Low Risk | High Risk |  |
|  |  |  |  |  |  |  | |
| Staikou et al 2014 | Some Concerns | Low Risk | Low Risk | Low Risk | Low Risk | Some Concerns | |
| Wongyingsinn et al 2011 | Low Risk | Some Concerns | Low Risk | Low Risk | Low Risk | Some Concerns | |
| Swenson et al 2010 | Low Risk | High Risk | Low Risk | Low Risk | Low Risk | High Risk | |
| Kuo et al 2006 | High Risk | Low Risk | Low Risk | Low Risk | Low Risk | High Risk | |

**Supplemental Table 3: a) RCTS**- Quality Assessment according to Revised Cochrane risk-of-bias tool for Randomized Trials (ROB2).

| **Study Name** | **Overall Score out of 9** |
| --- | --- |
| Terkawi et al 2016 | 5 |

**Supplemental Table 3: b) Observational Study**- Quality Assessment according to Newcastle Ottawa scale
