# Supplementary material for: Comparative Effectiveness of Epidural Analgesia and Intravenous Lidocaine for Postoperative Pain in Major Abdominal Surgery: A Systematic Review and Meta-Analysis
Source: Anesthesiol Res Pract. 2025 Feb 28;2025:9822744. doi: 10.1155/anrp/9822744 (PMC11991782; doi:10.1155/anrp/9822744)
Supplement: Supporting Information — Supporting Figure 1: Forest Plots of Mean Pain Scores (Sensitivity Analysis). (A): At 2 h Interval. (B): At 48 h Interval. [file 9822744.f4.pdf]

A

Study or Subgroup	Epidural Analgesia Mean	SD	Total	IV Lidocaine Mean	SD	Total	Weight	Std. Mean Difference IV, Random, 95% CI
**1.1.1 RCTs**								
Casas-Arroyave et al 2023	3.4	2.7	104	4.4	2.4	106	43.5%	-0.39 [-0.66, -0.12]
Jayaprabhu et al 2022	2	3.01	16	4	4.01	19	12.1%	-0.54 [-1.22, 0.13]
**Subtotal (95% CI)**			**120**			**125**	**55.5%**	**-0.41 [-0.67, -0.16]**
Heterogeneity: Tau² = 0.00; Chi² = 0.17, df = 1 (P = 0.68); I² = 0%								
Test for overall effect: Z = 3.19 (P = 0.001)								
**1.1.2 Observational Studies**								
Terkawi et al 2016	4.08	18.57	108	5.4	12.53	108	44.5%	-0.08 [-0.35, 0.18]
**Subtotal (95% CI)**			**108**			**108**	**44.5%**	**-0.08 [-0.35, 0.18]**
Heterogeneity: Not applicable								
Test for overall effect: Z = 0.61 (P = 0.54)								
**Total (95% CI)**			**228**			**233**	**100.0%**	**-0.27 [-0.53, -0.02]**
Heterogeneity: Tau² = 0.02; Chi² = 3.24, df = 2 (P = 0.20); I² = 38%								
Test for overall effect: Z = 2.10 (P = 0.04)								
Test for subgroup differences: Chi² = 3.07, df = 1 (P = 0.08), I² = 67.4%								

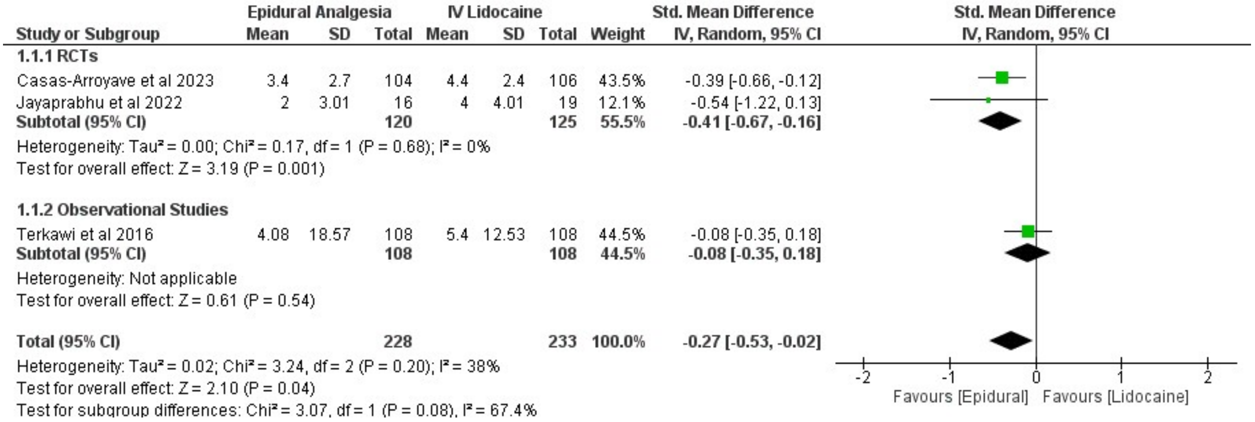

B

Study or Subgroup	Epidural Analgesia Mean	SD	Total	IV Lidocaine Mean	SD	Total	Weight	Std. Mean Difference IV, Random, 95% CI
**1.7.1 RCTs**								
Casas-Arroyave et al 2023	2.2	1.5	104	2.4	1.3	106	37.0%	-0.14 [-0.41, 0.13]
Kuo et al 2006	2	1	20	2.1	0.6	20	7.1%	-0.12 [-0.74, 0.50]
Swenson et al 2010	2.9	3.7	20	2.5	2	22	7.4%	0.13 [-0.47, 0.74]
Wongyingsinn et al 2011	0.7	1.6	30	1.5	2	30	10.3%	-0.44 [-0.95, 0.08]
**Subtotal (95% CI)**			**174**			**178**	**61.8%**	**-0.16 [-0.37, 0.05]**
Heterogeneity: Tau² = 0.00; Chi² = 2.05, df = 3 (P = 0.56); I² = 0%								
Test for overall effect: Z = 1.46 (P = 0.15)								
**1.7.2 Observational Studies**								
Terkawi et al 2016	3.6	8.3	108	3.5	10.4	108	38.2%	0.01 [-0.26, 0.28]
**Subtotal (95% CI)**			**108**			**108**	**38.2%**	**0.01 [-0.26, 0.28]**
Heterogeneity: Not applicable								
Test for overall effect: Z = 0.08 (P = 0.94)								
**Total (95% CI)**			**282**			**286**	**100.0%**	**-0.09 [-0.26, 0.07]**
Heterogeneity: Tau² = 0.00; Chi² = 2.97, df = 4 (P = 0.56); I² = 0%								
Test for overall effect: Z = 1.10 (P = 0.27)								
Test for subgroup differences: Chi² = 0.92, df = 1 (P = 0.34), I² = 0%								

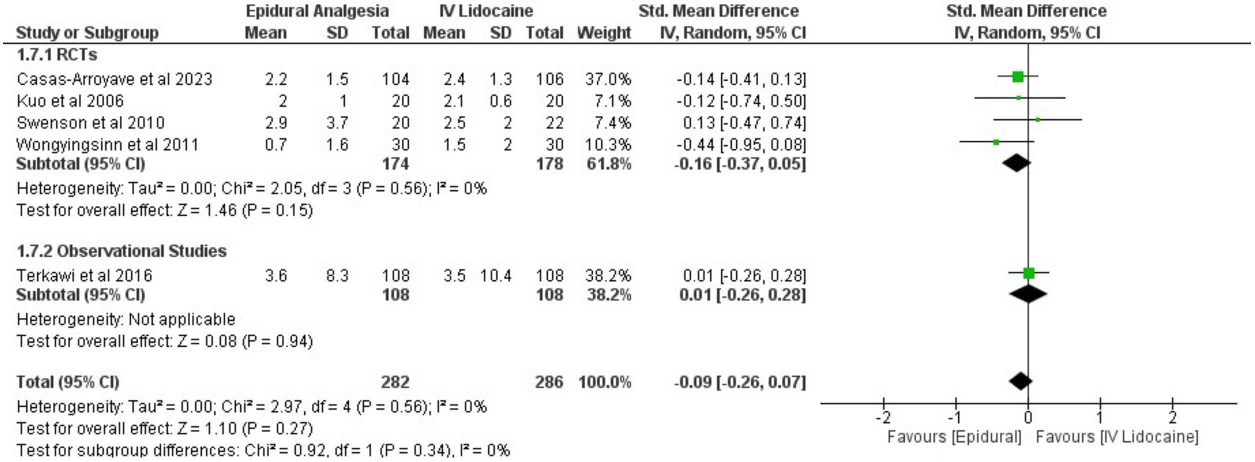

Supplemental Figure 1: Forest Plots of Mean Pain Scores (Sensitivity Analysis). A: At 2 hours Interval. B: At 48 hours Interval.
