# Supplementary material for: Comparative Effectiveness of Epidural Analgesia and Intravenous Lidocaine for Postoperative Pain in Major Abdominal Surgery: A Systematic Review and Meta-Analysis
Source: Anesthesiol Res Pract. 2025 Feb 28;2025:9822744. doi: 10.1155/anrp/9822744 (PMC11991782; doi:10.1155/anrp/9822744)
Supplement: Supporting Information — Supporting Figure 2: Forest Plots of Mean Pain Scores (Subgroup Analysis: Epidural Bupivacaine vs. Epidural Lidocaine). (A): At 2 h Interval. (B): At 12 h Interval. (C): At 24 h Interval. (D): At 48 h Interval. (E): At 72 h Interval. [file 9822744.f5.pdf]

A

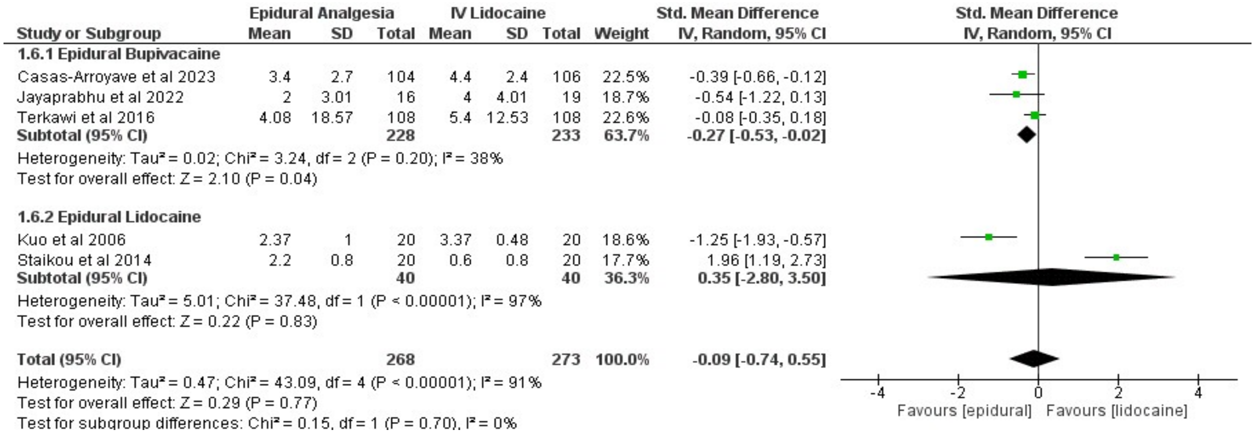

B

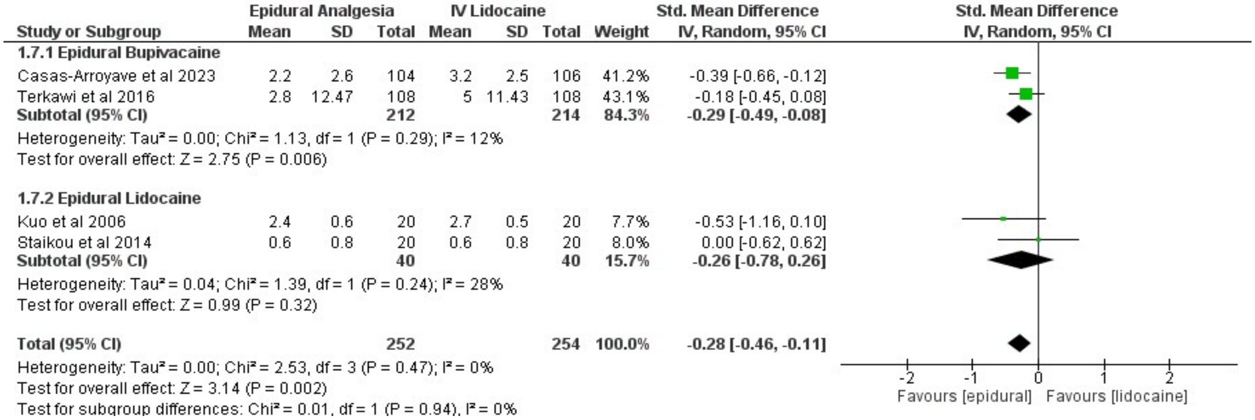

C

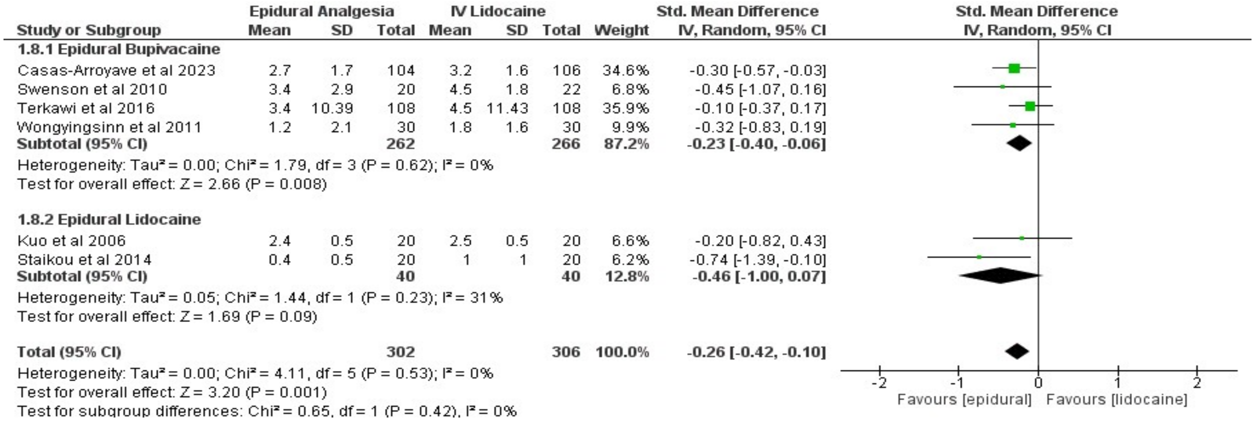

Supplemental Figure 2: Forest Plots of Mean Pain Scores (Subgroup Analysis: Epidural Bupivacaine versus Epidural Lidocaine). A: At 2 hours Interval. B: At 12 hours Interval. C: At 24 hours Interval. D: At 48 hours Interval. E: At 72 hours Interval.

D

Study or Subgroup	Epidural Analgesia Mean	SD	Total	IV Lidocaine Mean	SD	Total	Weight	Std. Mean Difference IV, Random, 95% CI
**1.9.1 Epidural Bupivacaine**								
Casas-Arroyave et al 2023	2.2	1.5	104	2.4	1.3	106	24.2%	-0.14 [-0.41, 0.13]
Swenson et al 2010	3	3.8	20	2.6	2	22	12.7%	0.13 [-0.48, 0.74]
Terkawi et al 2016	3.3	10.1	108	3.3	14.02	108	24.4%	0.00 [-0.27, 0.27]
Wongyingsinn et al 2011	0.7	1.6	30	1.5	2	30	15.3%	-0.44 [-0.95, 0.08]
**Subtotal (95% CI)**			**262**			**266**	**76.6%**	**-0.09 [-0.27, 0.08]**
Heterogeneity: Tau² = 0.00; Chi² = 2.84, df = 3 (P = 0.42); I² = 0%								
Test for overall effect: Z = 1.09 (P = 0.28)								
**1.9.2 Epidural Lidocaine**								
Kuo et al 2006	1.9	0.6	20	2	0.4	20	12.3%	-0.19 [-0.81, 0.43]
Staikou et al 2014	0.4	0.5	20	1.2	0.8	20	11.1%	-1.18 [-1.85, -0.50]
**Subtotal (95% CI)**			**40**			**40**	**23.4%**	**-0.67 [-1.64, 0.29]**
Heterogeneity: Tau² = 0.37; Chi² = 4.40, df = 1 (P = 0.04); I² = 77%								
Test for overall effect: Z = 1.37 (P = 0.17)								
**Total (95% CI)**			**302**			**306**	**100.0%**	**-0.24 [-0.52, 0.04]**
Heterogeneity: Tau² = 0.06; Chi² = 12.06, df = 5 (P = 0.03); I² = 59%								
Test for overall effect: Z = 1.67 (P = 0.10)								
Test for subgroup differences: Chi² = 1.35, df = 1 (P = 0.25), I² = 25.8%								

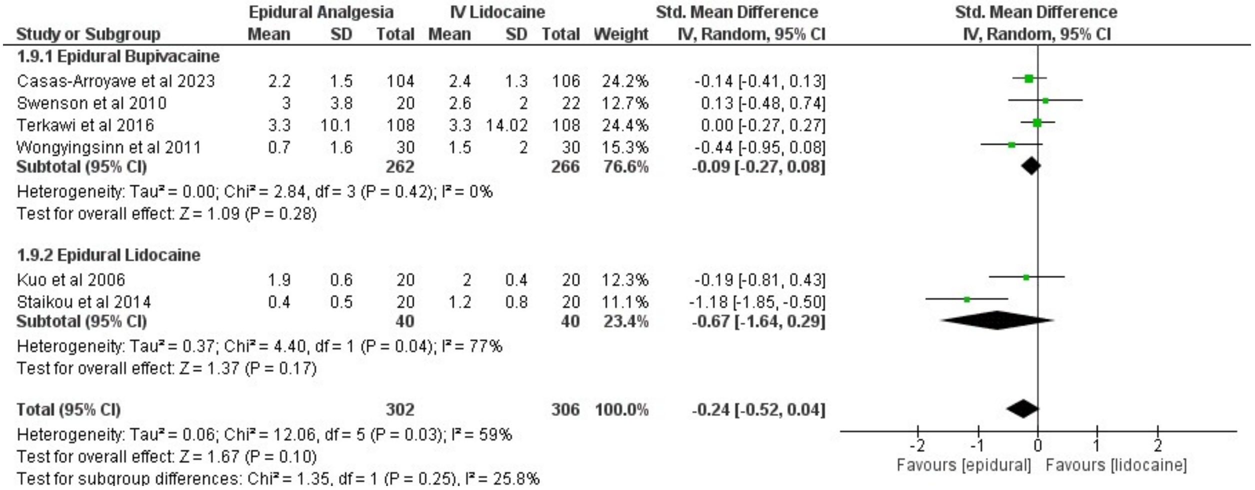

E

Study or Subgroup	Epidural Analgesia Mean	SD	Total	IV Lidocaine Mean	SD	Total	Weight	Std. Mean Difference IV, Random, 95% CI
**1.10.1 Epidural Bupivacaine**								
Casas-Arroyave et al 2023	1.8	1.3	104	1.9	1.2	106	37.0%	-0.08 [-0.35, 0.19]
Swenson et al 2010	1.9	1.6	20	1.9	1.9	22	7.4%	0.00 [-0.61, 0.61]
Terkawi et al 2016	2.8	9.8	108	3.7	13.2	108	38.1%	-0.08 [-0.34, 0.19]
Wongyingsinn et al 2011	1.1	1.6	30	1.3	1.3	30	10.6%	-0.14 [-0.64, 0.37]
**Subtotal (95% CI)**			**262**			**266**	**93.0%**	**-0.08 [-0.25, 0.09]**
Heterogeneity: Tau² = 0.00; Chi² = 0.11, df = 3 (P = 0.99); I² = 0%								
Test for overall effect: Z = 0.90 (P = 0.37)								
**1.10.2 Epidural Lidocaine**								
Kuo et al 2006	1.5	0.5	20	1.4	0.5	20	7.0%	0.20 [-0.43, 0.82]
**Subtotal (95% CI)**			**20**			**20**	**7.0%**	**0.20 [-0.43, 0.82]**
Heterogeneity: Not applicable								
Test for overall effect: Z = 0.62 (P = 0.54)								
**Total (95% CI)**			**282**			**286**	**100.0%**	**-0.06 [-0.22, 0.11]**
Heterogeneity: Tau² = 0.00; Chi² = 0.81, df = 4 (P = 0.94); I² = 0%								
Test for overall effect: Z = 0.71 (P = 0.48)								
Test for subgroup differences: Chi² = 0.70, df = 1 (P = 0.40), I² = 0%								

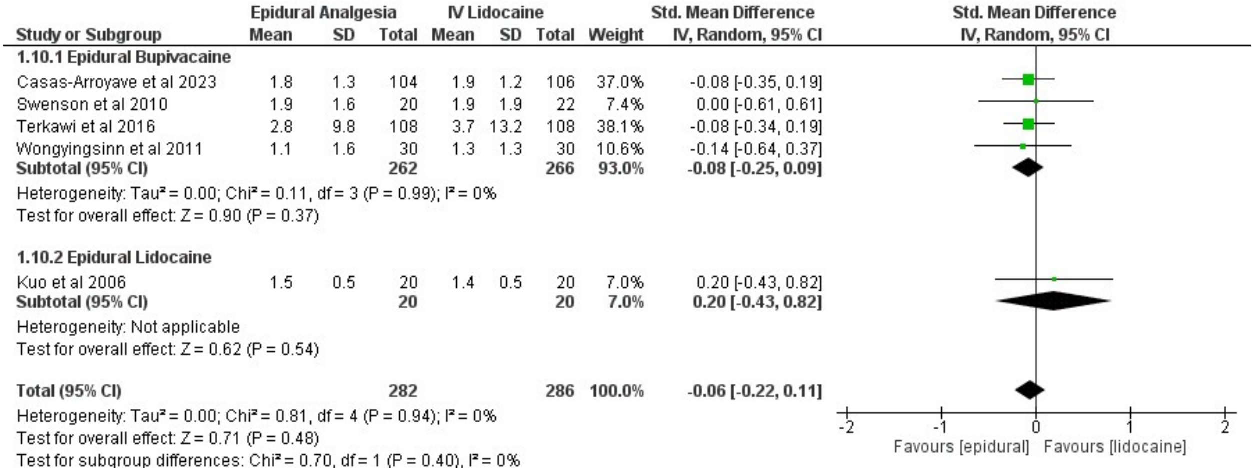

Supplemental Figure 2: Continued.
