# Supplementary material for: Comparative Effectiveness of Epidural Analgesia and Intravenous Lidocaine for Postoperative Pain in Major Abdominal Surgery: A Systematic Review and Meta-Analysis
Source: Anesthesiol Res Pract. 2025 Feb 28;2025:9822744. doi: 10.1155/anrp/9822744 (PMC11991782; doi:10.1155/anrp/9822744)
Supplement: Supporting Information — Supporting Figure 3: Forest Plots of Mean Daily Morphine Consumption equivalent to IV mg of morphine (Sensitivity Analysis). (A): At 24 h Interval. (B): At 48 h Interval. (C): At 72 h Interval. [file 9822744.f6.pdf]

A

Study or Subgroup	Epidural Analgesia Mean	SD	Total	IV Lidocaine Mean	SD	Total	Weight	Mean Difference IV, Random, 95% CI
**1.1.1 RCTs**								
Casas-Arroyave et al 2023	3.3	3.6	104	5.5	6	106	99.1%	-2.20 [-3.54, -0.86]
Swenson et al 2010	28.9	31	20	27.7	37.8	22	0.4%	1.20 [-19.63, 22.03]
**Subtotal (95% CI)**			**124**			**128**	**99.5%**	**-2.19 [-3.52, -0.85]**
Heterogeneity: Tau² = 0.00; Chi² = 0.10, df = 1 (P = 0.75); I² = 0%								
Test for overall effect: Z = 3.22 (P = 0.001)								
**1.1.2 Observational Studies**								
Terkawi et al 2016	10.5	55.1	108	21.9	85.2	108	0.5%	-11.40 [-30.54, 7.74]
**Subtotal (95% CI)**			**108**			**108**	**0.5%**	**-11.40 [-30.54, 7.74]**
Heterogeneity: Not applicable								
Test for overall effect: Z = 1.17 (P = 0.24)								
**Total (95% CI)**			**232**			**236**	**100.0%**	**-2.23 [-3.56, -0.90]**
Heterogeneity: Tau² = 0.00; Chi² = 0.99, df = 2 (P = 0.61); I² = 0%								
Test for overall effect: Z = 3.29 (P = 0.001)								
Test for subgroup differences: Chi² = 0.89, df = 1 (P = 0.35), I² = 0%								

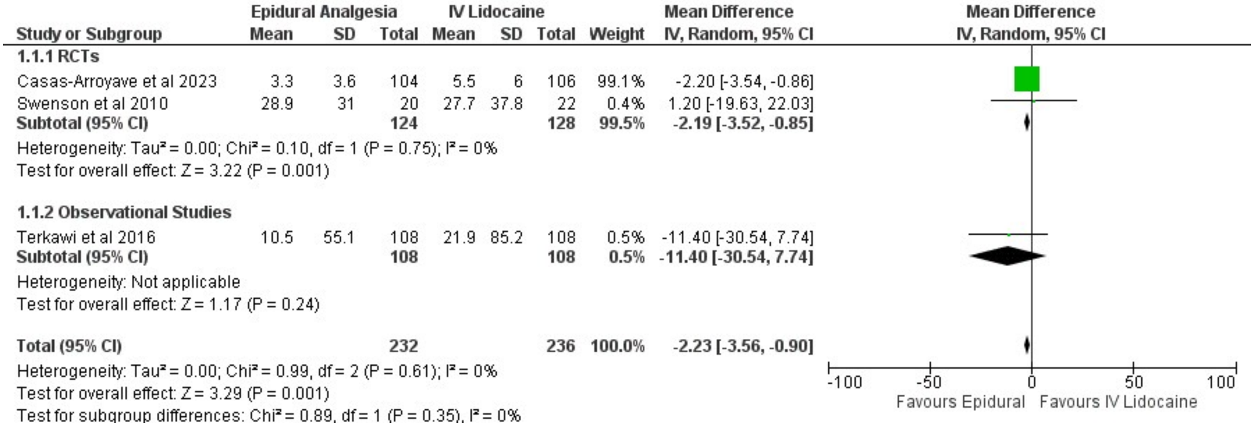

B

Study or Subgroup	Epidural Analgesia Mean	SD	Total	IV Lidocaine Mean	SD	Total	Weight	Mean Difference IV, Random, 95% CI	Year
**1.2.1 RCTs**									
Swenson et al 2010	61.7	58	20	54	41.8	22	0.2%	7.70 [-23.14, 38.54]	2010
Casas-Arroyave et al 2023	3.4	4	104	7.2	6	106	99.2%	-3.80 [-5.18, -2.42]	2023
**Subtotal (95% CI)**			**124**			**128**	**99.4%**	**-3.78 [-5.15, -2.40]**	
Heterogeneity: Tau² = 0.00; Chi² = 0.53, df = 1 (P = 0.47); I² = 0%									
Test for overall effect: Z = 5.38 (P < 0.00001)									
**1.2.2 Observational Studies**									
Terkawi et al 2016	12	74.8	108	15.8	62.3	108	0.6%	-3.80 [-22.16, 14.56]	2016
**Subtotal (95% CI)**			**108**			**108**	**0.6%**	**-3.80 [-22.16, 14.56]**	
Heterogeneity: Not applicable									
Test for overall effect: Z = 0.41 (P = 0.68)									
**Total (95% CI)**			**232**			**236**	**100.0%**	**-3.78 [-5.15, -2.41]**	
Heterogeneity: Tau² = 0.00; Chi² = 0.53, df = 2 (P = 0.77); I² = 0%									
Test for overall effect: Z = 5.40 (P < 0.00001)									
Test for subgroup differences: Chi² = 0.00, df = 1 (P = 1.00), I² = 0%									

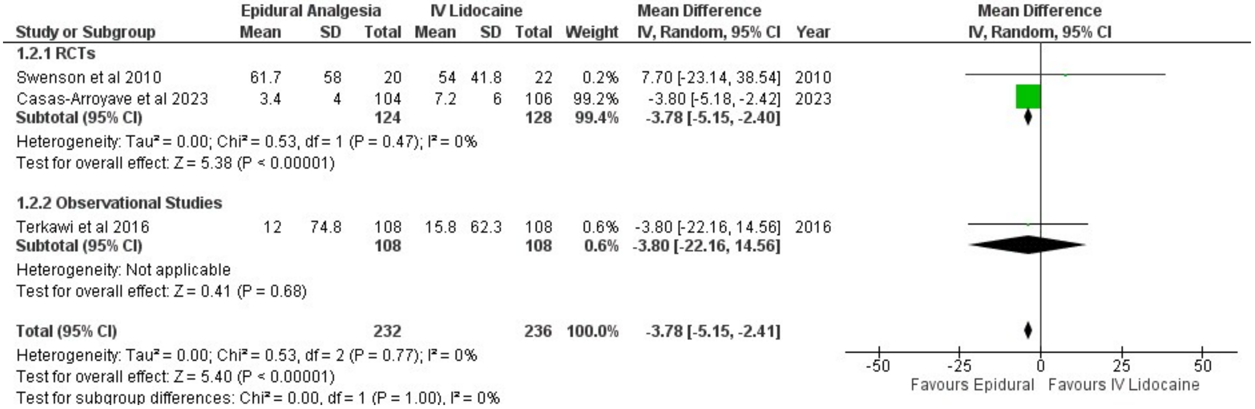

C

Study or Subgroup	Epidural Analgesia Mean	SD	Total	IV Lidocaine Mean	SD	Total	Weight	Mean Difference IV, Random, 95% CI	Year
**1.3.1 RCTs**									
Swenson et al 2010	51.9	42	20	39	46.5	22	0.2%	12.90 [-13.87, 39.67]	2010
Casas-Arroyave et al 2023	3.4	4	104	7	5.3	106	99.4%	-3.60 [-4.87, -2.33]	2023
**Subtotal (95% CI)**			**124**			**128**	**99.6%**	**-0.99 [-12.79, 10.82]**	
Heterogeneity: Tau² = 42.67; Chi² = 1.46, df = 1 (P = 0.23); I² = 31%									
Test for overall effect: Z = 0.16 (P = 0.87)									
**1.3.2 Observational Studies**									
Terkawi et al 2016	10.9	74.8	108	14.7	72.7	108	0.4%	-3.80 [-23.47, 15.87]	2016
**Subtotal (95% CI)**			**108**			**108**	**0.4%**	**-3.80 [-23.47, 15.87]**	
Heterogeneity: Not applicable									
Test for overall effect: Z = 0.38 (P = 0.70)									
**Total (95% CI)**			**232**			**236**	**100.0%**	**-3.56 [-4.83, -2.30]**	
Heterogeneity: Tau² = 0.00; Chi² = 1.46, df = 2 (P = 0.48); I² = 0%									
Test for overall effect: Z = 5.52 (P < 0.00001)									
Test for subgroup differences: Chi² = 0.06, df = 1 (P = 0.81), I² = 0%									

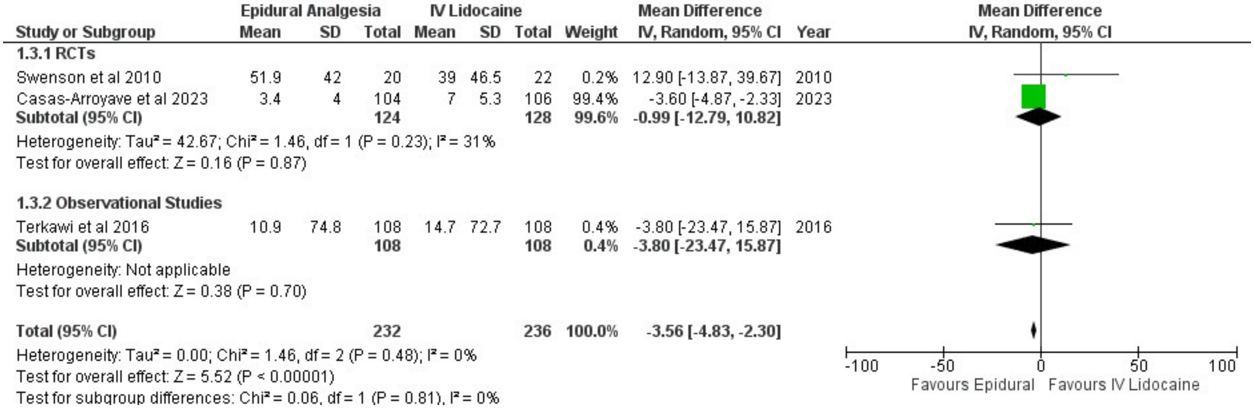

Supplemental Figure 3: Forest Plots of Mean Daily Morphine Consumption equivalent to IV mg of morphine (Sensitivity Analysis). A: At 24 hours Interval. B: At 48 hours Interval. C: At 72 hours Interval.
