# Supplementary material for: Comparative Effectiveness of Epidural Analgesia and Intravenous Lidocaine for Postoperative Pain in Major Abdominal Surgery: A Systematic Review and Meta-Analysis
Source: Anesthesiol Res Pract. 2025 Feb 28;2025:9822744. doi: 10.1155/anrp/9822744 (PMC11991782; doi:10.1155/anrp/9822744)
Supplement: Supporting Information — Supporting Figure 4: Forest Plots of Mean time to pass first flatus in hours (Sensitivity Analysis). [file 9822744.f7.pdf]

Study or Subgroup	Epidural Analgesia Mean	SD	Total	IV Lidocaine Mean	SD	Total	Weight	Mean Difference IV, Random, 95% CI
Kuo et al 2006	50.2	4.9	20	60.2	5.8	20	48.4%	-10.00 [-13.33, -6.67]
Staikou et al 2014	65.6	11.2	20	72.4	6.5	20	26.6%	-6.80 [-12.48, -1.12]
Swenson et al 2010	50.5	42.1	20	64.8	30.4	22	2.4%	-14.30 [-36.70, 8.10]
Wongyingsinn et al 2011	24.3	11.7	30	27.3	13.4	30	22.6%	-3.00 [-9.37, 3.37]
**Total (95% CI)**			**90**			**92**	**100.0%**	**-7.67 [-11.20, -4.14]**

Heterogeneity: $Tau^2 = 3.82$; $Chi^2 = 4.21$, $df = 3$ ($P = 0.24$); $I^2 = 29\%$

Test for overall effect: $Z = 4.26$ ($P < 0.0001$)

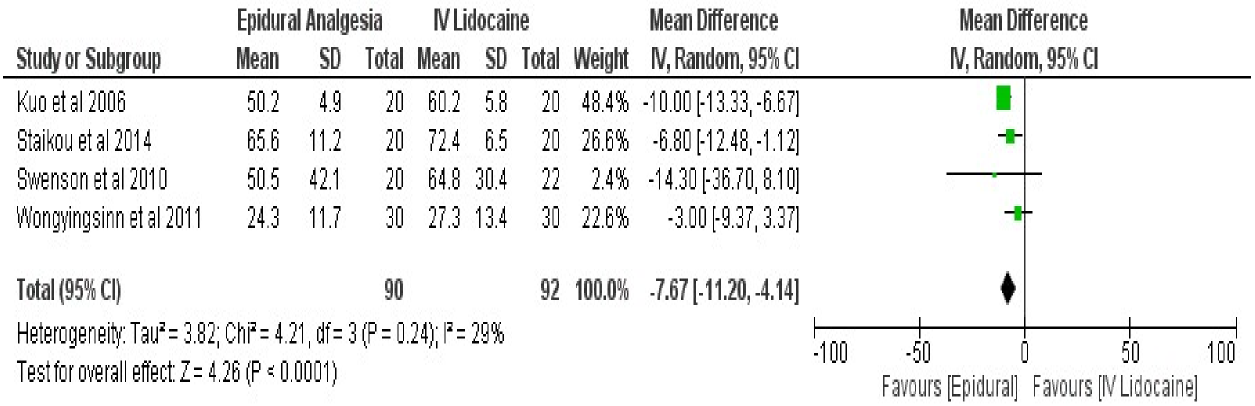

Supplemental Figure 4: Forest Plots of Mean time to pass first flatus in hours. (Sensitivity Analysis).
