# Supplementary figures and images for: Comparative Effectiveness of Epidural Analgesia and Intravenous Lidocaine for Postoperative Pain in Major Abdominal Surgery: A Systematic Review and Meta-Analysis
Source: Anesthesiol Res Pract. 2025 Feb 28;2025:9822744. doi: 10.1155/anrp/9822744 (PMC11991782; doi:10.1155/anrp/9822744)

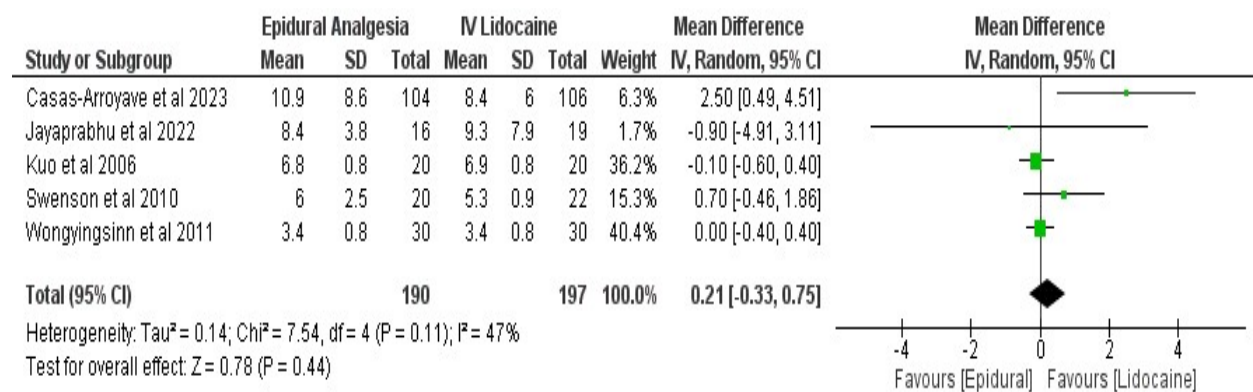

**Supplemental Figure 5:** Forest Plots of Mean Hospital Duration in days. (Sensitivity Analysis).

Supplement: Supporting Information — Supporting Figure 5: Forest Plots of Mean Hospital Duration in days (Sensitivity Analysis). [file 9822744.f8.pdf]
